# Ephrin Ligands and Eph Receptors Show Regionally Restricted Expression in the Developing Palate and Tongue

**DOI:** 10.3389/fphys.2016.00060

**Published:** 2016-02-23

**Authors:** Guilherme M. Xavier, Isabelle Miletich, Martyn T. Cobourne

**Affiliations:** ^1^Department of Craniofacial Development and Stem Cell Biology, King's College London Dental Institute, Guy's HospitalLondon, UK; ^2^Department of Orthodontics, King's College London Dental Institute, Guy's HospitalLondon, UK

**Keywords:** palatogenesis, tongue development, ephrin, Eph, gene expression, *in situ* hybridization

## Abstract

The Eph family receptor-interacting (ephrin) ligands and erythropoietin-producing hepatocellular carcinoma (Eph) receptors constitute the largest known family of receptor tyrosine kinases. Ephrin ligands and their receptors form an important cell communication system with widespread roles in normal physiology and disease pathogenesis. In order to investigate potential roles of the ephrin-Eph system during palatogenesis and tongue development, we have characterized the cellular mRNA expression of family members *EphrinA1-*A3, *EphA1*–*A8*, and *EphrinB2, EphB1, EphB4* during murine embryogenesis between embryonic day 13.5–16.5 using radioactive *in situ* hybridization. With the exception of *EphA6* and *ephrinA3*, all genes were regionally expressed during the process of palatogenesis, with restricted and often overlapping domains. Transcripts were identified in the palate epithelium, localized at the tip of the palatal shelves, in the mesenchyme and also confined to the medial epithelium seam. Numerous Eph transcripts were also identified during tongue development. In particular, *EphA1* and *EphA2* demonstrated a highly restricted and specific expression in the tongue epithelium at all stages examined, whereas *EphA3* was strongly expressed in the lateral tongue mesenchyme. These results suggest regulatory roles for ephrin-EphA signaling in development of the murine palate and tongue.

## Introduction

**Figure 1:**
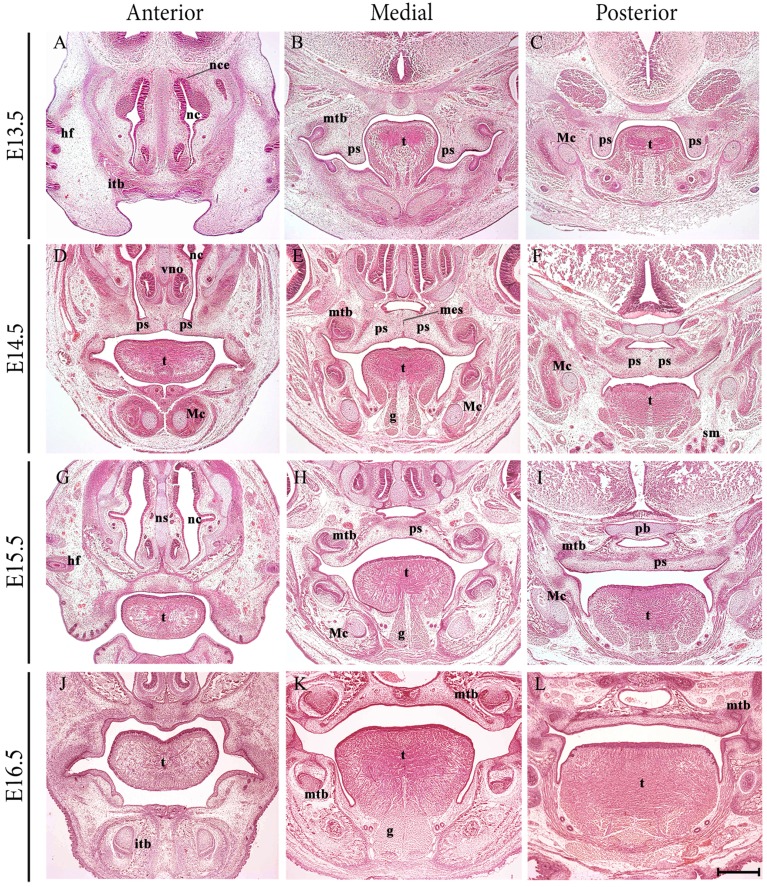
**Frontal sections through the developing craniofacial region of the early mouse embryo between E13.5 and E16.5**. At E13.5, the palatal shelves are positioned vertically adjacent to the developing tongue. At E14.5 the shelves have elevated above the tongue and grown to meet their counterpart at the midline, where the layers of epithelium adhere and begin to fuse with each other. At E15.5, continuity has been achieved and the palate separates the oral and nasal cavities. At E16.5, palatogenesis is essentially complete. g, genioglossus; hf, hair follicle; itb, incisor tooth bud; Mc, Meckel's cartilage; mes, medial epithelium seam; mtb molar tooth bud; nc, nasal cavity; ns, nasal septum; pb, presphenoid bone; ps, palatal shelves; sm, submandibular gland; t, tongue. Scale bar in L = 500 μm for **(A–L)**.

The formation of a palate separating the oral and nasal cavities is a developmental process characteristic of higher vertebrates and requires complex and highly coordinated molecular interactions (reviewed in Ferguson, 1988; Cobourne, 2004; Dudas et al., 2007; Gritli-Linde, 2007). In the embryo, the primary palate is a derivative of the frontonasal process, whilst the secondary palate forms from the paired palatal shelves of the maxillary process, themselves a derivative of the first pharyngeal arch. The palate is formed by elevation and fusion of the maxillary palatal shelves, with each other posteriorly, with the primary palate anteriorly and the nasal septum superiorly (reviewed in Dudas et al., 2007). The palatal structures are built from cranial neural crest (CNC)-derived ectomesenchyme, mesoderm and the oro-pharyngeal ectoderm (reviewed in Ferguson, 1988). In mice, the palate is formed relatively late in organogenesis, with the palatal shelves initially appearing at embryonic day (E) 11.5 and growing vertically adjacent to the developing tongue from E12.5 to E14.0. However, by E14.5 the shelves have elevated above the tongue and grown to meet their counterpart at the midline, where the layers of epithelium adhere and then fuse with each other to achieve continuity in the roof of the oral cavity (Figures 1A–L).

Palatal shelf elevation is a rapid process, accompanied and facilitated by changes within the extracellular matrix of the palatal shelf mesenchyme and the coordinated movement of other craniofacial structures. It is generally accepted that elevation of the palatal shelves above the tongue and their associated change in orientation from a vertical to horizontal position, arises from a combination of intrinsic and extrinsic forces, including descent of the tongue (Ferguson, 1988). The complexity of palatogenesis means that in humans it is frequently disturbed, resulting in the birth defect of cleft palate (reviewed in Ferguson, 1988; Cobourne, 2004). The causes of cleft palate as a malformation can be broadly categorized on an embryological basis as a lack of adequate growth in the palatal shelves, failure to elevate above the tongue or a breakdown in the mechanism of fusion between the shelves. In addition, cleft palate can also arise secondary to other craniofacial malformations, such as micrognathia and basoccipital or basisphenoid fusion, craniosynostosis and both muscle and tongue abnormalities (reviewed in Ferguson, 1988; Rice et al., 2004; Casey et al., 2006; Chai and Maxson, 2006; Gritli-Linde, 2007; Xiong et al., 2009).

Development of the vertebrate tongue involves contributions from CNC cells derived from pharyngeal arches 1–3 and the somitic myoblasts (Parada and Chai, 2015). The oral portion or anterior two thirds of the murine tongue emerges from the floor of the early oral cavity as a set of mesenchymal swellings derived from the first branchial arch. A medial lingual swelling initially forms, but this is rapidly engulfed by two lateral lingual swellings that will form the anterior two thirds proper. The posterior third or pharyngeal component is derived from two further swellings within the third branchial arch, the copula and hypopharyngeal eminence (Noden and Francis-West, 2006; Hosokawa et al., 2010). In the mouse embryo, the process of tongue development begins around E10.5, with a noticeable tongue bud evident by E12.5, which undergoes rapid enlargement and differentiation to form a large muscular organ by E16.5 (Parada et al., 2012; Figures 1A–L).

The Eph family receptor-interacting (ephrin) ligands and erythropoietin-producing hepatocellular carcinoma (Eph) receptors have been extensively studied since their discovery (Hirai et al., 1987). Ephs constitute the largest known family of receptor tyrosine kinases, comprising at least 16 distinct receptors that are highly conserved (Hirai et al., 1987; Jones et al., 1995; Scales et al., 1995; Lackmann and Boyd, 2008; Islam et al., 2010). Based on structural features in their ligand-binding domains and their ephrin-binding preferences, Ephs are classified into 10 EphA and 6 EphB receptors. The EphA group preferentially bind glycosylphosphatidylinositol (GPI)-linked ligands of the ephrin-A subclass; whilst the EphB group preferentially interact with transmembrane ligands of the ephrin-B subclass (reviewed in Lackmann and Boyd, 2008). However, EphA4 binds both classes of ephrin and EphB2 can bind ephrinA5 (Himanen et al., 2004; Dravis and Henkemeyer, 2011).

Together, Eph receptors and their ligands, form an important cell communication system with widespread roles in normal physiology and disease pathogenesis (Pasquale, 2005, 2010). Eph–ephrin complexes emanate bidirectional signals, forward signals that depend on Eph kinase activity propagated in the receptor-expressing cell and reverse signals, that depend on Src family kinases propagated in the ephrin-expressing cell. Ephrin-dependent but kinase-independent Eph signals can also occur (Gu and Park, 2001; Matsuoka et al., 2005; Miao et al., 2005). Eph signaling is known to control cell morphology, adhesion, migration, and invasion by modifying organization of the actin cytoskeleton and influencing the activities of integrins and intercellular adhesion molecules (Pasquale, 2005, 2010; Klein, 2012).

There is evidence from both humans and mice for the potential involvement of specific ephrin and Eph family members during palate development. In the human craniofrontonasal syndrome, mutations in *EPHRINB1* give rise to a range of cranial defects, including cleft lip and palate (Twigg et al., 2004; Wieland et al., 2004; Torii et al., 2007); whilst targeted disruption of *EphrinB1* in mice results in craniofacial and other skeletal defects, including cleft palate (Orioli et al., 1996; Compagni et al., 2003; Davy et al., 2004). Additionally, engineering of compound transgenic mice for *EphB2* and *EphB3* leads to cleft palate; suggesting that a combination of EphB3 protein and EphB2 forward signaling is important for palate development (Risley et al., 2009).

EphA-family receptor expression patterns have previously been described in the developing palate (Agrawal et al., 2014); however, only limited tongue expression data was shown. On the basis of this previous data and a rudimentary PCR-based screen of EphA transcriptional activity in the developing palate (data not shown) we have investigated expression of EphA-family members and their ephrin-A ligands during murine palate and tongue development. We also mapped *ephrinb2* expression in these regions, given that this ligand interacts with EphA4, and ephrinB2 reverse signaling is known to be important for normal closure of the secondary palate (Dravis and Henkemeyer, 2011). In addition, *EphB1* and *EphB4* expression was analyzed. EphB1 has also been associated with cleft lip and palate in human populations (Watanabe et al., 2006) and previously identified as the preferred receptor of ephrinB2 in the mechanism of axonal pathfinding (Chenaux and Henkemeyer, 2011); whilst EphB4 only binds ephrinB2 amongst all the ephrin-B family ligands (Sakano et al., 1996).

We find widespread expression of these family members during murine palatogenesis. In addition, regionally-restricted expression of many members in the developing tongue, suggests some commonalities during the coordinated development of the palate and tongue.

## Materials and methods

Mouse plasmids containing cDNA were linearized with the appropriate restriction enzymes and antisense ^35^S-UTP radio-labeled riboprobes generated using specific RNA polymerases (Table 1).

**Table 1:** **Plasmids used for the generation of riboprobes**.

**Gene**	**Source**	**Sequence length**	**Restriction enzyme**	**Polymerase**
*ephrinA1*	David Wilkinson	1.5 kb	SalI	T3
*ephrinA2*	David Wilkinson	1.6 kb	HindIII	T3
*ephrinA3*	David Wilkinson	1.2 kb	NotI	T7
*EphA1*	RZPD-IMAGE 4196138	3.2 kb—Full length	EcorV	T7
*EphA2*	David Wilkinson	1.3 kb—3′ coding	HindIII	T7
*EphA3*	Tyler Cutforth	1.7 kb—extracell dom	XBaI	T3
*EphA4*	David Wilkinson	1.5 kb—3′UTR	HindIII	T7
*EphA5*	Andrea Ballabio	0.6 kb—3′UTR	EcoRI	T3
*EphA6*	David Feldheim	4.4 kb	BamHI	T7
*EphA7*	RZPD-IMAGE 3991628	3.5 kb—Full length	EcoRV	T7
*EphA8*	Tyler Cutforth	0.5 kb	EcorV	T3
*ephrinB2*	Andrea Ballabio	1 kb—Full length ORF	NotI	T3
*EphB1*	Mark Henkemeyer	0.6 kb—Exon 3	SacII	SP6
*EphB4*	David Anderson	1 kb—kinase frag	EcoRI	T7

CD-1 mice were time-mated and pregnant females sacrificed with cervical dislocation. Matings were set up such that noon of the day on which vaginal plugs were detected was considered as E0.5. Embryos were collected between E13.5 and E16.5, fixed in 4% (w/v) paraformaldehyde at 4°C overnight, washed in PBS, dehydrated through a graded series of ethanols, embedded in paraffin wax and sectioned at 7 μm, prior to section *in situ* hybridisation.

Radioactive section *in situ* hybridisation was carried out as previously described (Xavier et al., 2009). Light and dark-field images of sections were photographed using a Zeiss Axioscop microscope and merged in Adobe Photoshop CS2.

## Results and discussion

**Figure 2:**
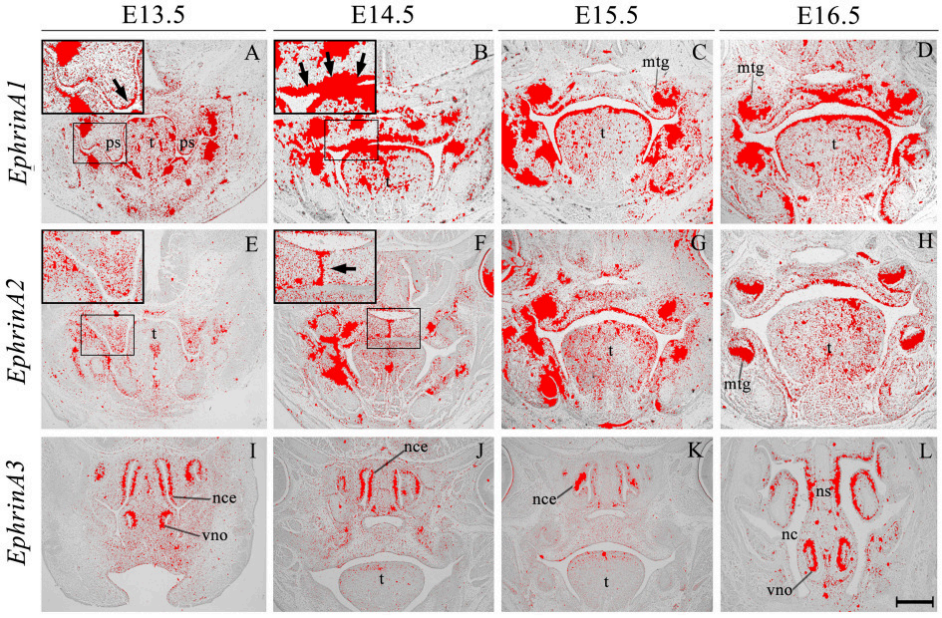
***EphrinA1*, *EphrinA2, and EphrinA3* expression in the craniofacial region of the mouse embryo between E13.5 and E16.5**. Radioactive *in situ* hybridization of frontal sections of embryos. Regions of *EphrinA1* and *EphrinA2* expression in the palatal shelf are highlighted at E13.5 and E14.5, respectively in **(A, E)** and **(B, F)** for each gene. At E13.5, *EphrinA1* expression was intensified in epithelium at the tip of the palatal shelf (arrow in **A**), and strongly expressed throughout the epithelium at E14.5 (arrow in **B**). *EphrinA2* was localized to the medial epithelial seam of the palate during fusion of the shelves at E14.5 (arrow in **F**). *EphrinA3* expression was confined to epithelium of the developing nasal cavity and vomeronasal organ as shown in sections **(I–L)**. mtg, molar tooth germ; nc, nasal cavity; nce, nasal cavity epithelium; ns, nasal septum; ps, palatal shelves; t, tongue; vno, vomeronasal organ. Scale bar in L = 500 μm for **(A–L)**.

*EphrinA1* transcripts were identified in the palate epithelium from E13.5 to E16.5 (Figures 2A–D), particularly at the tip of the palatal shelf at E13.5 (Figure 2A, highlighted) with strong expression throughout the oral surface of the palatal shelf epithelium at E14.5 (Figure 2B, highlighted). In contrast, *EphrinA1* was only expressed at background levels between E13.5 and E16.5 (Figures 2A–D). *EphrinA2* showed no specific epithelial expression in the palate at E13.5, although transcripts were present in the mesenchyme (Figure 2E, highlighted); however, by E14.5 distinct transcriptional activity was observed in the MES (Figure 2F, highlighted). During subsequent development at E15.5–E16.5 *EphrinA2* upregulated in the palatal shelf epithelium (Figures 2G,H). *EphrinA3* was not detected above low-level background signal in the developing palate between E13.5 and E16.5 (data not shown). However, at E13.5 transcripts were identified in epithelium of the developing vomeronasal organ and nasal cavity (Figure 2I), expression domains that were maintained between E14.5 and E16.5 (Figures 2J–L).

**Figure 3:**
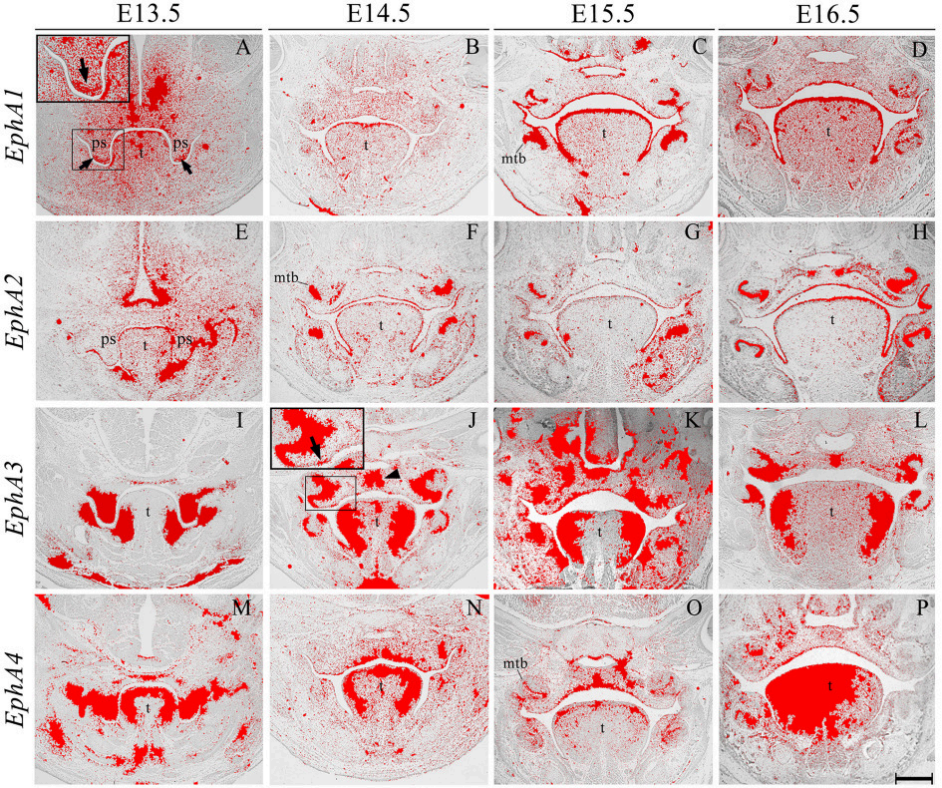
***EphA1*, *EphA2*, *EphA3*, and *EphA4* expression in the craniofacial region of the mouse embryo between E13.5 and E16.5**. Radioactive *in situ* hybridization of frontal sections of embryos. Regions of *EphA1* and *EphA3* expression are highlighted at E13.5 and E14.5 in **(A,J)**, respectively. At E13.5, *EphA1* expression was intensified in mesenchyme at the tip of the palatal shelf (arrows in **A**). *EphA3* expression was localized to regions of the epithelium and a broad region of epithelium and mesenchyme across the midline of the palate during fusion at E14.5 (arrow and arrowhead in **J**, respectively). *EphA4* expression was strong in the palatal shelves prior to elevation **(M)**, and localized to the oral epithelium and MES at later stages **(N–P)**. mtb, molar tooth bud; ps, palatal shelves; t, tongue. Scale bar in P = 500 μm for **(A–P)**.

*EphA1* was generally expressed in the palatal shelf mesenchyme at E13.5, and in a complementary manner to its ligand EphrinA1 (see Figure 2A), was upregulated in mesenchyme at the tip of the shelves (Figure 3A, highlighted). Lower-level expression was maintained in the palatal mesenchyme at later stages (Figures 3B–D), but at E15.5–E16.5 *EphA1* was clearly upregulated in the oral epithelium after palatal shelf fusion (Figures 3C,D). In contrast, no expression was detected in epithelium of the MES (Figure 3B), which is in agreement with previous findings (Agrawal et al., 2014). *EphA2* was detected in the palatal shelf epithelium from E13.5–E16.5 (Figures 3E–H); although no transcriptional activity was observed in the MES (Figure 3F). *EphA2* has been shown to function as a positive regulator of mammary epithelial proliferation and branching (Vaught et al., 2009; Park J. E. et al., 2013) and it is known that growth of the palatal shelves is controlled by reciprocal epithelial-mesenchymal interactions along the antero-posterior axis (Bush and Jiang, 2012; Economou et al., 2013). Based on the distinctive expression pattern within the epithelium, *EphA2* may be important for normal growth of the early palatal shelves.

**Figure 4:**
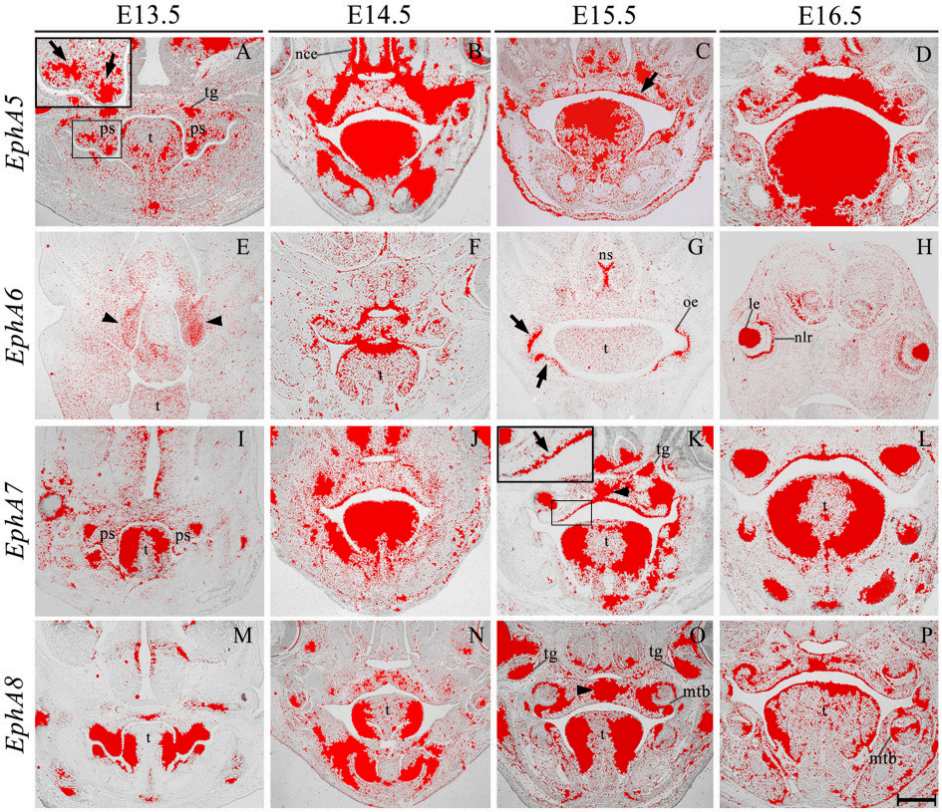
***EphA5*, *EphA6*, *EphA7*, and *EphA8* expression in the craniofacial region of the mouse embryo between E13.5 and E16.5**. Radioactive *in situ* hybridization of frontal sections of embryos. Regions of *EphA5* and *EphA7* expression are highlighted at E13.5 and E15.5 in **(A,K)**, respectively. At E13.5, there was patchy expression of *EphA5* in the palatal shelf mesenchyme (arrow in **A**), whilst at E15.5, expression was detected in the palatal shelf epithelium (arrow in **C**). *EphA6* expression was confined to mesenchyme of the developing nasal cavity at E13.5 (arrowheads in **E**) but upregulated in oral epithelium at E15.5 (arrows in **G**). *EphA7* was strongly expressed in the epithelium throughout palatogenesis, but was upregulated in the palatal midline at E15.5 (arrow and arrowhead in **K**, respectively). *EphA8* was also strongly expressed in the mid-palatal region at E15.5 (arrowhead in **O**). le, lens; mtb, molar tooth bud; nce, nasal cavity epithelium; nlr, neural layer of the retina; oe, oral epithelium; ps, palatal shelves; t, tongue; tg, trigeminal ganglion. Scale bar in P = 500 μm for **(A–P)**.

*EphA3* was intensely expressed throughout the palatal shelves at E13.5 (Figure 3I), although this expression became localized to regions of epithelium at E14.5 (Figure 3J, highlighted) in contrast to previous observations, transcripts were also detected in the midline during the process of fusion, including the MES and regions of adjacent mesenchyme (Figure 3J, arrowhead). *EphA3* remained enriched in these regions of the palate epithelium and mesenchyme during subsequent stages of palatogenesis between E15.5 and E16.5 (Figures 3K,L; Agrawal et al., 2014). *EphA4* was also strongly expressed throughout the palatal shelves prior to elevation at E13.5 (Figure 3M), progressively localizing to the oral epithelium and MES during later development (Figures 3N–P). Despite this dynamic expression pattern, an absence of both *EphA3* and *EphA4* function does not result in any overt developmental phenotype in the mouse, including the palate. Redundant roles played by other family members may explain the lack of palate phenotype in compound *EphA3*^−∕−^; *EphA4*^−∕−^ mutant embryos (Agrawal et al., 2014). *EphA5* hybridization signals were present in a patchy distribution within the mesenchymal component of the palatal shelves at E13.5, (Figure 4A, highlight); whilst during later stages, expression was detected throughout the epithelium and very strongly in mesenchyme at the lateral edges of the palate, with this strong expression also observed in the nasal cavity epithelium (Figure 4B). Following fusion at E15.5, *EphA5* was localized to the palatal epithelium (Figure 4C, arrowed); however, at E16.5, marked up-regulation was observed in the mesenchyme, but restricted to medial regions of the fused shelves (Figure 4D). *EphA6* transcripts were not detected at any significant level in the palatal shelves at E13.5 (data not shown) although some upregulation was seen in mesenchyme of the nasal cavity (Figure 4E, arrowheads). Transcripts were detected in palatal epithelium of the oral cavity during fusion at E14.5, but they were absent from the MES (Figure 4F) and no expression was observed following fusion of the palatal shelves at E15.5 (Figure 4G) and E16.5 (data not shown). Interestingly, there was strong localized expression of *EphA6* in epithelium of the oral commissure at E15.5 (Figure 4G, arrowed) and intense expression also identified in the lens and neural layer of the retina at E16.5 (Figure 4H). *EphA7* was consistently detected in the palatal shelf epithelium throughout palatogenesis, but only weakly in the mesenchyme (Figures 4I–L, highlight in Figure 4K). However, at E15.5 strong midline expression was detected (Figure 4K, arrowhead). This expression pattern was different from that described in previously published data, where *EphA7* was mainly observed in the mesenchyme (Agrawal et al., 2014). *EphA8* showed intense expression in both the epithelium and mesenchyme of the palatal shelves at E13.5 (Figure 4M), with lower-level expression at later stages; again, with the exception at E15.5, where strong expression was detected in the midline mesenchymal region (Figures 4N–P, arrowhead in Figure 4O), which also differs from that previously reported (Agrawal et al., 2014). Recently, *in vivo* expression of EphA8-Fc was reported to result in neuroepithelial cell apoptosis and a subsequent decrease in brain size (Kim et al., 2013). These findings are in agreement with previous studies that demonstrated that Ephrin-Eph signaling plays a critical role in determining the size of the neuroepithelial cell population during early embryonic brain development (Holmberg et al., 2000; Park E. et al., 2013). *EphA8* may therefore have a role in mediating epithelial apoptosis during the process of palatal shelf fusion.

**Figure 5:**
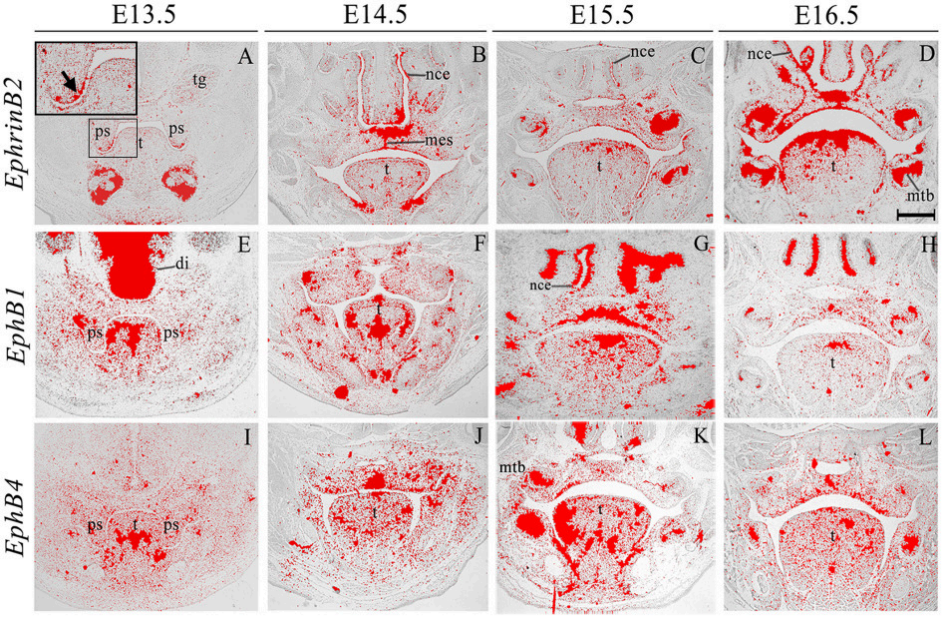
***EphrinB2, EphB1*, *EphB4* expression in the craniofacial region of the mouse embryo between E13.5 and E16.5**. Radioactive *in situ* hybridization of frontal sections of embryos. *EphrinB2* expression is highlighted in **A** (arrowed). di, diencephalon; mes, medial epithelium seam; mtb, molar tooth bud; nce, nasal cavity epithelium; ps, palatal shelves; t, tongue; tg, trigeminal ganglion. Scale bar in D = 500 μm for **(A–L)**.

*EphrinB2* was expressed in the epithelium and (more weakly) in the mesenchyme during palatogenesis, particularly at the tip of the palatal shelves at E13.5 and in the MES at E14.5 (Figures 5A–D, highlighted in Figure 5A). *EphB1* transcriptional activity was weak but widespread in the palatal shelf mesenchyme at E13.5–E14.5; however, by E15.5 expression was up-regulated in the midline of the embryonic palate, returning to previous levels by E16.5 (Figures 5E–H). *EphB4* was also weakly expressed in the palatal shelf mesenchyme throughout palatogenesis, but with strong midline expression at E14.5 in the MES during shelf fusion (Figures 5I–L, arrow in Figure 5J). *EphB1* expression has been previously reported in the venous vasculature throughout embryonic development to adulthood (Li and Mukouyama, 2013). Additionally, *EphB1* has been observed in the mouse retina (Birgbauer et al., 2000), during the early stages of embryonic rat spinal cord development (Jevince et al., 2006) and in the basal ganglia nuclei (Richards et al., 2007). Interestingly, behavioral evaluation of *EphB1* null mice in an open-field environment has revealed the presence of spontaneous locomotor hyperactivity (Richards et al., 2007). During palatogenesis streams of directional cell migration (both in the anterior and posterior aspect) have been demonstrated to occur and are thought to be of importance for palate patterning (shaping) and elevation (He et al., 2008). Interestingly, a cellular migration system solely dependent on EphrinB2–EphB4 signal transduction has demonstrated that EphB4 is capable of triggering the regulation of cell migration (Sturz et al., 2004). Taken together, these results suggest that these genes could also be involved in cell migration events that take place during palate development.

**Figure 6:**
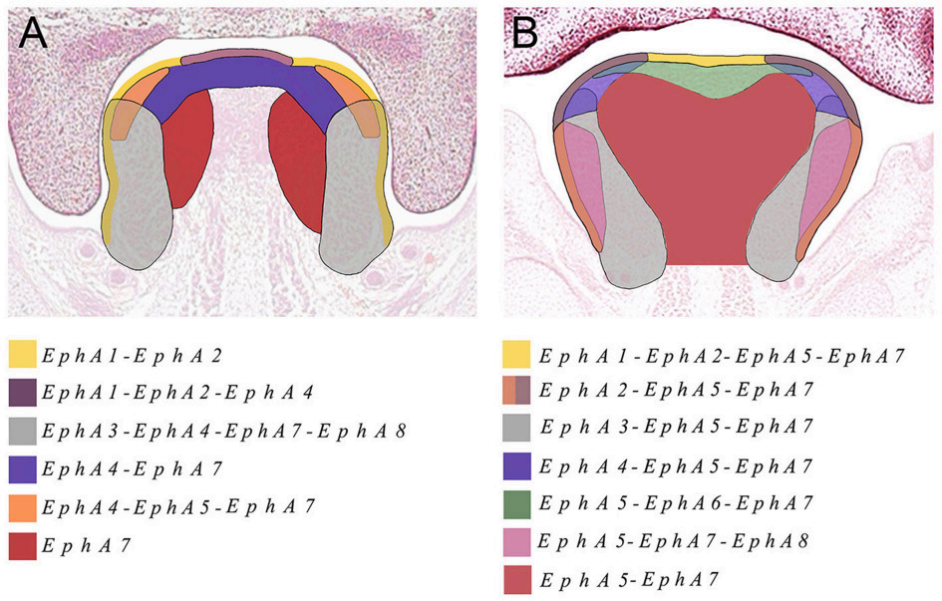
**The main domains of expression associated with the EphA family of receptors in the developing tongue at (A) E13.5 and (B) E14.5. Diagrams not drawn to scale**.

We also identified the expression of numerous Ephs during murine tongue development between E13.5 and E16.5. The main domains of expression associated with the EphA group in the tongue at E13.5 and E14.5 are summarized in Figures 6A,B. *EphA5* and *EphA7* presented with ubiquitous expression at E14.5 (see Figures 4B,J, respectively). During all stages examined, *EphA1* and *EphA2* demonstrated distinctive expression in the tongue epithelium (Figures 3A–H), whereas *EphA3* was strongly expressed in the lateral tongue mesenchyme between E13.5 and E16.5 (Figures 3I–L). Although *EphA4* was also detected in the lateral tongue mesenchyme at earlier stages (Figures 3M,N); by E15.5, transcriptional activity was down-regulated (Figure 3O) and restricted to patchy regions of the epithelium (Figure 3O), although at E16.5, expression was increased in the mesenchyme (Figure 3P). *EphA6* presented weak and widespread expression in the mesenchyme during tongue development (Figures 3E–G). However, at E14.5, a marked upregulation was observed in the inter-molar eminence of the tongue (Figure 4F). Similarly to *EphA3, EphA7*, and *EphA8* transcriptional activity were also markedly increased in the lateral mesenchyme of the tongue during development (Figures 4I–O). However, by E16.5 *EphA8* expression was down-regulated and more restricted to the epithelial compartment (Figure 4P). Rapid depression of the tongue in embryogenesis is critical for proper palatogenesis. Any delay in this process can disturb palatal shelf elevation and hence, lead to cleft palate (Nie, 2005). For these events to take place a coordinated balance between apoptosis and proliferation is essential (Parada et al., 2012; Parada and Chai, 2015).

**Figure 7:**
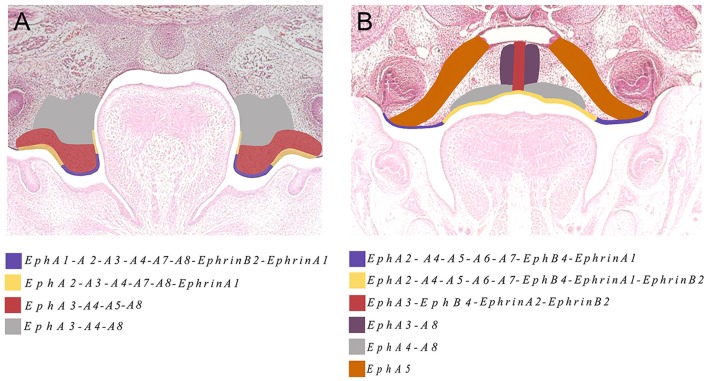
**The main domains of expression associated with ephrins and Eph receptors in the developing palate at (A) E13.5 and (B) E14.5. Diagrams not drawn to scale**.

The Eph and ephrin family-member gene expression in the developing palate and tongue described here is summarized in Figures 6, 7. These dynamic domains suggest important potential roles for these molecules in both epithelium and mesenchyme during development of these regions. Further analysis using animal models will be required to delineate the precise requirements during these developmental processes. However, the co-expression of *EphA3, A4*, and *A8* in the palatal shelves makes it difficult to test the hypothesis that these genes are involved in palatogenesis. Considering the known promiscuous interactions between Ephs and ephrins, it is likely EphA3, A4, and A8 may also play redundant roles during palate development. Analysis of a triple loss-of-function mouse model may be required to definitively address this question.

## Conclusions

Eph receptors A3, A4, and A8 are very strongly expressed within palatal shelf mesenchyme during early palatogenesis and both EphA1 and A5 are up-regulated at the shelf tip during this stage. Eph receptors A3, A4, and A8 are also strongly expressed in lateral regions of the tongue at these stages, suggesting some co-ordination in the regulation of palatogenesis and tongue development. EphA and ephrinA-family members are also expressed in palatal shelf epithelium (*EphA2, EphA7, ephrinA1*) and mesenchyme (*EphA1, A3, A4, A5, A6, A8*, and *ephrinA2, A3*) suggesting the possibility of epithelial-mesenchymal interactions being mediated by these proteins during development of the palate.

## Author contributions

MC and IM conceived the experiments, GX and IM conducted the experiments and undertook data acquisition, GX, IM, and MC wrote the manuscript.

### Conflict of interest statement

The authors declare that the research was conducted in the absence of any commercial or financial relationships that could be construed as a potential conflict of interest.
